# Diagnostics and new treatment regimens for TB: can the Xpert MTB/XDR assay fill the gap for fluoroquinolone testing?

**DOI:** 10.1128/jcm.00643-25

**Published:** 2025-09-03

**Authors:** Derek T. Armstrong, Emma C. E. Baird, Logan Pretty, Karen D’Agostino, Matthew Schwartz, Victoria L. Campódonico, Nicole Parrish

**Affiliations:** 1 The Johns Hopkins Medical Systems, Baltimore, Maryland, USA; University of Manitoba, Winnipeg, Manitoba, Canada

**Keywords:** drug resistance, tuberculosis, diagnostics, mycobacteria

## Abstract

Rapid diagnosis of resistance-conferring mutations to antibiotics used for the treatment of tuberculosis (TB) is critical for patient care and public health control efforts. Prior guidelines included the use of fluoroquinolones (FQs) for the treatment of drug-resistant TB, including multidrug-resistant TB, pre-extensively drug-resistant TB, and extensively drug-resistant TB. More recently, a short-course regimen for antibiotic-susceptible TB was introduced, which includes the use of a FQ, a drug class that diagnostic algorithms in the United States (US) typically do not test for if all first-line agents are susceptible. However, FQ mono-resistance has been documented by previous studies, and for this reason, we tested 319 archived *Mycobacterium tuberculosis* complex (MTBC) strains spanning a 14-year period of time using the Xpert MTB/XDR assay. Resistance to FQs was detected in 4.4% (14/319) of the isolates tested, with mutations predominating in the *gyrA* region (13/14; 92.9%). A single isolate (1/14; 7.1%) was found to have a *gyrB* mutation. A broth microdilution assay demonstrated the minimum inhibitory concentrations for resistant strains that ranged from 0.5 µg/mL to 8.0 µg/mL. Importantly, three strains were FQ mono-resistant and would have been completely missed by standard testing algorithms. Although currently unavailable in the US, the GeneXpert XDR assay has the potential to fill the significant diagnostic gap in susceptibility testing of MTBC resistance to FQs and support the use of the currently recommended short-course regimen.

**IMPORTANCE:**

This study provides insight into the need for additional rapid testing for the detection of drug resistance (specifically to fluoroquinolones) in tuberculosis (TB) cases in the United States (US). The current regimens for TB treatment rely on knowing resistance patterns to optimize treatment, and missed resistance could have a negative impact on the health of the patient, as well as contribute to increased drug-resistance mutations in new TB cases. There are currently limited platforms for expanded rapid drug resistance testing for TB cases in the US, and this study looks at past TB cases that had drug resistance missed by routine testing.

## INTRODUCTION

Tuberculosis (TB) remains a significant public health concern in the United States (US). Following the SARS-CoV-2 pandemic, TB cases increased from 8,320 in 2022 to 9,615 in 2023 (1). This increase of 16% was noted among all age groups and was seen in both US- and non-US-born individuals, with more than 8,900 cases reported in 2020. Despite efforts to control the disease, the emergence of drug-resistant TB strains poses a significant challenge to TB control programs in the country. One critical issue requiring urgent attention in the US is the need for more up-to-date diagnostic tools for the detection of antimicrobial resistance in TB laboratories to support current treatment regimens.

Current diagnostic algorithms in the US for the detection and identification of the *Mycobacterium tuberculosis* complex (MTBC) from clinical specimens have relied on both traditional laboratory methods including microscopy, culture, and phenotypic drug-susceptibility testing (pDST), as well as newer molecular methods (Xpert MTB/RIF, Cepheid, Sunnyvale, CA) (2–5). However, the need for change in these algorithms recently emerged with the discontinuation of the AccuProbe assay (Hologic, Newark, DE) for identification of the MTBC from positive cultures and the endorsement by the U.S. Center for Disease Control (CDC) of a short-course treatment regimen for drug-susceptible TB. For identification, many laboratories have opted to replace the AccuProbe assay with either the Xpert MTB/RIF assay or a lab-developed nucleic acid amplification-based assay (6). However, questions remain for many laboratories regarding susceptibility testing in support of the short-course treatment regimen. This regimen, which is used to treat drug-susceptible TB, consists of rifapentine, isoniazid, pyrazinamide, and moxifloxacin daily for 8 weeks followed by an additional 9 weeks of the same drugs minus pyrazinamide (7). Data supporting the use of this regimen were obtained through an international Phase 3 CDC-sponsored clinical trial, which demonstrated that the treatment of drug-susceptible TB could be achieved in 4 months instead of the standard 6 months (7). This short-course regimen provides more rapid treatment and will also aid in improving patient compliance. Most clinical mycobacteriology laboratories in the US currently perform pDST for all of the standard first-line drugs, including rifampin, isoniazid, ethambutol, and pyrazinamide. It has been argued that rifampin DST results can serve as a surrogate for rifapentine, since both are from the same class of drug. However, moxifloxacin is not currently tested by most laboratories in the US, as up until now, it was considered a second-line agent. This includes both pDST and genotypic DST (gDST) methods. Compounding the diagnostic dilemma, the World Health Organization (WHO) recently changed the definition of pre-extensively drug-resistant (pre-XDR)- and extensively drug-resistant (XDR)-TB, in which traditional second-line injectable drugs have been completely removed, such that pre-XDR-TB now refers to multidrug-resistant TB (MDR-TB; resistant to isoniazid and rifampin) or rifampin-resistant TB (RR-TB) plus resistance to any FQ (8). XDR-TB has been redefined as pre-XDR-TB plus resistance to at least bedaquiline, linezolid, or both drugs (8).

As a result, a significant gap in overall laboratory DST currently exists with regard to the FQs, including moxifloxacin, which could lead to the use of this drug in the short-course regimen without confirmation of susceptibility or resistance. This is especially important given that prior studies have shown rates of FQ resistance as high as 11% following a relatively short exposure to this class of drug, some of which involve treatment for other infectious diseases where TB is an unidentified co-morbidity (9, 10).

Recently, the Xpert MTB/XDR assay (Cepheid) was introduced in 2021 to more rapidly diagnose drug-resistant TB globally (11–13). This platform utilizes the same technology as the Xpert MTB/RIF assay, detecting genotypic drug resistance within 2 h. However, unlike the Xpert MTB/RIF assay, which detects resistance only to rifampin, the GeneXpert XDR assay can identify resistance to multiple antibiotics, including isoniazid, second-line injectable drugs, ethionamide, and most importantly, the FQs (11–13).

To better understand the scope of genotypic FQ resistance in TB strains previously characterized over the last 14 years at our institution, we undertook a retrospective research study using the Xpert MTB/XDR platform to evaluate the utility of rapidly detecting FQ resistance for patients being considered for the short-course regimen.

## MATERIALS AND METHODS

Clinical MTBC strains utilized in this study came from the Johns Hopkins Clinical Mycobacteriology research archive and were chosen randomly to include samples from both US-based cases and ex-US cases across a 14-year span (2009–2023). All isolates had previously been identified as *Mycobacterium tuberculosis* using the AccuProbe (Hologic) assay or the Xpert MTB/RIF (Cepheid). First-line drug-susceptibility testing was performed using the MGIT 960 system (Becton Dickinson, Sparks, Maryland). All isolates were previously de-identified to remove any demographic or protected health information and stored in 2 mL cryovial aliquots and stored at −80°C. Briefly, a sample of each isolate was removed from the −80°C freezer, and 1 mL was tested on the Xpert XDR platform according to the package insert (2:1 sample to reagent ratio). Testing personnel were blinded to any first-line drug-susceptibility results obtained with the MGIT 960 system. Test results that were indeterminate (either error or no result) were not repeated due to insufficient remaining sample volume. FQ resistance was identified by the detection of mutations found in the quinolone resistance-determining region containing the *gyrA* and *gyrB* genes, where a majority of known FQ mutations are found.

These FQ-resistant isolates were subsequently sub-cultured onto Lowenstein–Jensen media and incubated at 35°C in 5% CO_2_ until sufficient growth for pDST was obtained. Subsequently, the minimum inhibitory concentration (MIC) was determined for moxifloxacin for each strain using a broth microdilution test and established protocols (MYCOTB, ThermoFisher, Lenexa, Kansas). All assays were performed in duplicate, and MIC testing was performed by a separate technician who was not aware of the FQ resistance results from the Xpert MTB/XDR assay.

Testing occurred from 2021 to 2023 and included a total of 325 archived isolates from 2009 to 2023. All equipment, cartridges, and reagents were provided by Cepheid for the purpose of this study, and IRB approval was obtained at Johns Hopkins.

## RESULTS

A total of 325 tests were performed on the Xpert MTB/XDR platform, with 319/325 (98.2%) giving valid results where a majority of known FQ mutations are found, and 6/325 (1.8%) giving indeterminate results for MTBC identification. A final set of 319 isolate results was used for analysis, all of which were identified as MTBC by the Xpert MTB/XDR platform. Resistance to FQs was detected by the Xpert MTB/XDR platform in 14/319 (4.4%) isolates, with 1/14 (7.1%) showing a “Low Resistance” result. In strains for which FQ resistance was present, mutations in the *gyrA* region predominated in 13/14 (92.9%) isolates. A single isolate (1/14, 7.1%) was found to have a *gyrB* mutation. Table 1 provides a full overview of resistance to the FQs detected by the Xpert MTB/XDR assay and the corresponding pDST result showing the MIC for moxifloxacin obtained using a broth microdilution assay. Two isolates had moxifloxacin MICs of 0.5 μg/mL, one of which was reported as “Low Resistance Detected” to the FQs by the MTB/XDR assay. For all of the other strains, FQ “Resistance Detected” was reported by the MTB/XDR assay, where 6/14 (42.9%) had MICs of 2.0 µg/mL, 5/14 (35.7%) had MICs of 4.0 µg/mL, and one isolate had an MIC of 8.0 µg/mL. The category of each isolate as mono-resistant, MDR-TB, pre-XDR-TB, or XDR-TB, based on pDST and GeneXpert XDR results, is also shown in Table 1. Importantly, of the 14 strains for which FQ resistance was detected, 3 (21.4%) were completely susceptible to all first-line antibiotics, including rifampin, isoniazid, ethambutol, and pyrazinamide. These strains were also susceptible to all other drugs tested, including bedaquiline, clofazimine, pretomanid, linezolid, rifabutin, amikacin, and ethionamide. As such, these strains represent FQ mono-resistant isolates.

**TABLE 1:** MTBC strains for which gDST and pDST indicated the presence of fluoroquinolone resistance in this study^*a*^

MTBC strain #	pDST Resistance characterization	Xpert MTB/XDR FQ result	FQ mutation	MIC (µg/mL)
35	RIF mono-R	Resistance detected	*gyrA*	2.0
36	INH mono-R	Resistance detected	*gyrA*	2.0
70	INH mono-R	Resistance detected	*gyrA*	4.0
93	Pre-XDR	Resistance detected	*gyrA*	2.0
95	Pre-XDR	Resistance detected	*gyrA*	4.0
98	Pre-XDR	Resistance detected	*gyrA*	4.0
105	FQ mono-R	Resistance detected	*gyrA*	2.0
122	XDR	Resistance detected	*gyrA*	4.0
163	FQ mono-R	Resistance detected	*gyrA*	2.0
233	XDR	Low Resistance detected	*gyrA*	0.5
239	FQ mono-R	Resistance detected	*gyrA*	2.0
246	MDR	Resistance detected	*gyrA*	4.0
252	MDR	Resistance detected	*gyrB*	0.5
258	RIF mono-R	Resistance detected	*gyrA*	8.0

^*a*^ Abbreviations: gDST (genomic drug-susceptibility testing), pDST (phenotypic drug-susceptibility testing), FQ (fluoroquinolone), RIF (rifampicin), MDR (multidrug-resistant), XDR (extensively drug-resistant), MIC (minimum inhibitory concentration).

## DISCUSSION

In the current study, we undertook a retrospective approach using archived MTBC isolates at our institution to evaluate the utility of the Xpert MTB/XDR assay to rapidly detect fluoroquinolone (FQ) resistance and provide genotypic susceptibility guidance for patients being considered for the short-course regimen. Central to this regimen is the use of a FQ, including moxifloxacin, which is currently not tested by either pDST or gDST methods in the majority of US laboratories. In addition, FQs have, until recently, been considered a second-line agent, creating a diagnostic gap in susceptibility testing, since most US laboratories have been focused on testing of traditional first-line drugs: isoniazid, rifampin, ethambutol, and pyrazinamide.

Our study demonstrated that the Xpert MTB/XDR assay can detect FQ resistance not only in MDR-TB or XDR-TB strains but also in strains for which it was thought no resistance was present. These three strains, which were determined to be FQ mono-resistant, are of the most concern given the current susceptibility testing climate. For many laboratories performing only traditional first-line susceptibility testing, whether pDST or gDST, all would have missed the presence of FQ mono-resistance. This is especially important since previous studies have demonstrated that resistance to the fluoroquinolones develops relatively quickly in the MTBC upon exposure to the drug class in the absence of other antibiotics. In one study, it was demonstrated that exposure to FQ for at least 10 days or more prior to the diagnosis of TB correlated with a higher risk for development of resistance (14). Some recent reports have even suggested that FQ resistance is on the rise in the MTBC, despite being relatively stable in the past (15). In 2021, the WHO revised the definitions of pre-XDR-TB and XDR-TB, in which the former was defined as MDR-TB AND resistance to FQ, with the latter characterized as pre-XDR plus resistance to any of the Group “A” drugs, specifically bedaquiline and linezolid (8). In tandem with this is the WHO’s current recommendation for the use of a short-course FQ-containing regimen for the treatment of drug-susceptible TB for 4 months and RR-TB- or MDR-TB for 6 months (16). As a result, it is more important than ever to be able to rapidly identify resistance to the FQs in the MTBC through pDST and gDST methods. This is even more important given that many public health and other laboratories in the US do not routinely test the FQs if no resistance is seen for any of the first-line antibiotics.

This study has demonstrated that the Xpert MTB/XDR assay, if used as a triage test along with the Xpert MTB/RIF assay, could provide for rapid detection of FQ resistance not only in MDR-TB and pre-XDR-TB but also in the detection of FQ mono-resistant strains. In fact, the use of the Xpert MTB/XDR assay could provide a means for many laboratories to implement testing of this class of drug, especially in the US, where historically FQs were considered a second-line agent for the treatment of TB. This significant gap in diagnostic testing in the US will require the investment of resources to remedy, whether it be for a pDST or gDST solution. Commercial options are available for pDST in the Sensititre MYCOTB panel, as well as the BACTEC MGIT 960 system, although the latter is not available for distribution with this class of drugs in the US. Likewise, a line probe assay (Hain Lifescience, Nehren, Germany), which can also detect FQ mutations, is a high complexity test that can be problematic to obtain in the US. Thus, readily available solutions to this diagnostic deficit are limited at the current time with one exception. Many laboratories already have the requisite GeneXpert system in place, which would present key benefits if the XDR cartridge were available. The test can rapidly identify FQ-resistant strains, even directly from respiratory specimens, allowing clinicians to initiate appropriate treatment, including the 4- or 6-month short-course regimen, immediately. With the standard cartridge, which is already in use in the US, in tandem with the XDR cartridge, resistance to isoniazid, rifampin, and the FQs could be quickly assessed ahead of any confirmatory pDST that may be needed. This would be of significant diagnostic benefit, given that multiple studies, including our own, where 21.4% FQ mono-resistance was found, have demonstrated that mono-resistance to this class of drugs exists and is potentially being missed by many laboratories that do not perform further testing if all first-line drugs are susceptible. Although the number of FQ-resistant strains is not exceedingly high in the US population, it is important to recognize that they are not zero and, if encountered, would likely impact the newer short-course treatment regimens with potential detrimental effects on patient outcome and public health. Early initiation of appropriate treatment is essential in managing drug-resistant TB, and testing can support current treatment regimens by ensuring rapid and accurate detection of resistance.

This study has several limitations. Although first-line pDST was performed for all strains used in this study, FQ pDST (MICs; specifically moxifloxacin) was only determined for those isolates with detected resistance by the GeneXpert XDR assay. Since clinical strains from the JHU archive were used, the amount of FQ resistance, including mono-resistance to the drug class (or resistance to other FQ drugs, including levofloxacin), may not be representative of all regions. In addition, resistance determination to other drugs (including INH and RIF) was not analyzed in depth, in order to focus specifically on missed FQ resistance. Additional work should be undertaken to determine the impact of performing FQ testing as routine testing in the US, especially on clinical samples.

In conclusion, the use of the GeneXpert XDR assay in the US would provide a significant advancement in the diagnosis and management of drug-resistant TB, which is especially important given the short-course therapeutic recommendations issued by WHO. However, additional studies are needed to address the challenges of implementation since the assay is currently unavailable in the US. Should this change, the GeneXpert XDR assay has the potential to improve patient outcomes, prevent the spread of drug-resistant TB, and better support TB control efforts in the US.

## References

[1] Williams PM, Pratt RH, Walker WL, Price SF, Rebekah JS, Pei-Jean IF. 2024. Tuberculosis-United States, 2023. MMWR Morb Mortal Wkly Rep 73:265–270. doi:10.15585/mmwr.mm7312a4 38547024 PMC10986816

[2] Cepheid. 2015. Xpert MTB/RIF Assay*.* Sunnyvale, CA: Cepheid. Available from: http://www.cepheid.com/mtbrif

[3] Chaisson LH, Roemer M, Cantu D, Haller B, Millman AJ, Cattamanchi A, Davis JL. 2014. Impact of GeneXpert MTB/RIF assay on triage of respiratory isolation rooms for inpatients with presumed tuberculosis: a hypothetical trial. Clin Infect Dis 59:1353–1360. doi:10.1093/cid/ciu620 25091300 PMC4296131

[4] Lippincott CK, Miller MB, Popowitch EB, Hanrahan CF, Van Rie A. 2014. Xpert MTB/RIF assay shortens airborne isolation for hospitalized patients with presumptive tuberculosis in the United States. Clin Infect Dis 59:186–192. doi:10.1093/cid/ciu212 24729506 PMC4133562

[5] CDC. 2013. Availability of an assay for detecting Mycobacterium tuberculosis, including rifampin-resistant strains, and considerations for its use-United States, 2013. MMWR. Morbidity and Mortality Weekly Report. Vol. 62. https://www.cdc.gov/mmwr/preview/mmwrhtml/mm6241a1.htm. PMC4585342 24141407

[6] Armstrong DT, Fisher S, Totten M, Schwartz M, Gnanashanmugam D, Parrish N. 2022. Clinical validation of the Xpert MTB/RIF test for Identification of the Mycobacterium tuberculosis complex in acid-fast bacillus smear-positive MGIT broth cultures. J Clin Microbiol 60:e0216421. doi:10.1128/JCM.02164-21 34985982 PMC8849197

[7] Dorman SE, Nahid P, Kurbatova EV, Goldberg SV, Bozeman L, Burman WJ, Chang K-C, Chen M, Cotton M, Dooley KE, et al.. 2020. High-dose rifapentine with or without moxifloxacin for shortening treatment of pulmonary tuberculosis: study protocol for TBTC study 31/ACTG A5349 phase 3 clinical trial. Contemp Clin Trials 90:105938. doi:10.1016/j.cct.2020.105938 31981713 PMC7307310

[8] WHO. 2020. Meeting report of the WHO expert consultation on the definition of extensively drug-resistant tuberculosis. World Health Organization. https://www.who.int/publications/i/item/9789240018662.

[9] Ginsburg AS, Hooper N, Parrish N, Dooley KE, Dorman SE, Booth J, Diener-West M, Merz WG, Bishai WR, Sterling TR. 2003. Fluoroquinolone resistance in patients with newly diagnosed tuberculosis. Clin Infect Dis 37:1448–1452. doi:10.1086/379328 14614666

[10] Ginsburg AS, Sun R, Calamita H, Scott CP, Bishai WR, Grosset JH. 2005. Emergence of fluoroquinolone resistance in Mycobacterium tuberculosis during continuously dosed moxifloxacin monotherapy in a mouse model. Antimicrob Agents Chemother 49:3977–3979. doi:10.1128/AAC.49.9.3977-3979.2005 16127087 PMC1195434

[11] Xie YL, Chakravorty S, Armstrong DT, Hall SL, Via LE, Song T, Yuan X, Mo X, Zhu H, Xu P, et al.. 2017. Evaluation of a rapid molecular drug-susceptibility test for tuberculosis. N Engl J Med 377:1043–1054. doi:10.1056/NEJMoa1614915 28902596 PMC5727572

[12] Centner CM, Munir R, Tagliani E, Rieß F, Brown P, Hayes C, Dolby T, Zemanay W, Cirillo DM, David A, Schumacher SG, Denkinger CM, Ruhwald M, Leukes VN, Nicol MP, Van der Walt I, Kisten G, Gumede M, Mace A, Brink A, Stevens W, Scott L, Penn-Nicholson A, Cox H, TB-CAPT Consortium. 2024. Reflex Xpert MTB/XDR testing of residual rifampicin-resistant specimens: a clinical laboratory-based diagnostic accuracy and feasibility study in South Africa. Open Forum Infect Dis 11:ofae437. doi:10.1093/ofid/ofae437 39165581 PMC11334068

[13] Cepheid. 2021. Xpert MTB/XDR assay. Sunnyvale, CA: Cepheid. Available from: http://www.cepheid.com/mtbxdr-pi

[14] Devasia RA, Blackman A, Gebretsadik T, Griffin M, Shintani A, May C, Smith T, Hooper N, Maruri F, Warkentin J, Mitchel E, Sterling TR. 2009. Fluoroquinolone resistance in Mycobacterium tuberculosis: the effect of duration and timing of fluoroquinolone exposure. Am J Respir Crit Care Med 180:365–370. doi:10.1164/rccm.200901-0146OC 19483111 PMC2731810

[15] Ferran E, Chan C, Sheikh N, Dedicoat M, Alexander E, Gibertoni-Cruz A, Brown J, Robinson E, Lipman M. 2025. Population-level frequency of fluoroquinolone resistance by whole-genome sequencing drug predictions in Mycobacterium tuberculosis complex isolates in England from 2017 through 2023. Clin Infect Dis 80:660–662. doi:10.1093/cid/ciae560 39535753 PMC11912959

[16] World Health Organization. 2022. WHO operational handbook on tuberculosis module 4: treatment-drug susceptible tuberculosis treatment. Available from: https://www.who.int/publications/i/item/9789240050761 35727905

